# Prevalence, Risk Factors, and Treatment Outcomes of Isoniazid- and Rifampicin- Mono-Resistant Pulmonary Tuberculosis in Lima, Peru

**DOI:** 10.1371/journal.pone.0152933

**Published:** 2016-04-05

**Authors:** Leonela Villegas, Larissa Otero, Timothy R. Sterling, Moises A. Huaman, Patrick Van der Stuyft, Eduardo Gotuzzo, Carlos Seas

**Affiliations:** 1 Vanderbilt University School of Medicine, Nashville, TN, United States of America; 2 Instituto de Medicina Tropical Alexander von Humboldt, Universidad Peruana Cayetano Heredia, Lima, Peru; 3 Unit of General Epidemiology and Disease Control, Department of Public Health, Institute of Tropical Medicine, Antwerp, Belgium; 4 Department of Public Health, Faculty of Medicine, Ghent University, Ghent, Belgium; Texas A&M University, UNITED STATES

## Abstract

**Background:**

Isoniazid and rifampicin are the two most efficacious first-line agents for tuberculosis (TB) treatment. We assessed the prevalence of isoniazid and rifampicin mono-resistance, associated risk factors, and the association of mono-resistance on treatment outcomes.

**Methods:**

A prospective, observational cohort study enrolled adults with a first episode of smear-positive pulmonary TB from 34 health facilities in a northern district of Lima, Peru, from March 2010 through December 2011. Participants were interviewed and a sputum sample was cultured on Löwenstein-Jensen (LJ) media. Drug susceptibility testing was performed using the proportion method. Medication regimens were documented for each patient. Our primary outcomes were treatment outcome at the end of treatment. The secondary outcome included recurrent episodes among cured patients within two years after completion of the treatment.

**Results:**

Of 1292 patients enrolled, 1039 (80%) were culture-positive. From this subpopulation, isoniazid mono-resistance was present in 85 (8%) patients and rifampicin mono-resistance was present in 24 (2%) patients. In the multivariate logistic regression model, isoniazid mono-resistance was associated with illicit drug use (adjusted odds ratio (aOR) = 2.10; 95% confidence interval (CI): 1.1–4.1), and rifampicin mono-resistance was associated with HIV infection (aOR = 9.43; 95%CI: 1.9–47.8). Isoniazid mono-resistant patients had a higher risk of poor treatment outcomes including treatment failure (2/85, 2%, p-value<0.01) and death (4/85, 5%, p<0.02). Rifampicin mono-resistant patients had a higher risk of death (2/24, 8%, p<0.01).

**Conclusion:**

A high prevalence of isoniazid and rifampicin mono-resistance was found among TB patients in our low HIV burden setting which were similar to regions with high HIV burden. Patients with isoniazid and rifampicin mono-resistance had an increased risk of poor treatment outcomes.

## Introduction

Tuberculosis (TB) has been one of the leading infectious agents worldwide throughout the past century, but drug resistance has emerged more recently as a major concern [1–4]. The prevalence of mono-resistance to isoniazid, one of the most potent first-line anti-TB agents, has been reported in ranges from 4–12% for all TB cases with a global average of 8.1% for new TB cases [3–5]. There is less evidence for rifampicin mono-resistance because it is less studied, but prevalences under 1% for new TB cases have been reported within Europe in 2010 and 3.2% in Zambia [6, 7]. Meanwhile, multidrug-resistant TB (MDR-TB; defined as resistance to at least isoniazid and rifampicin) continues to be an extensive problem, with an estimated 3.5% of new cases globally in 2014 [4]. The optimal treatment of drug-resistant TB is unclear due to limited data from randomized, controlled trials, with a focus on aggressive regimens [8–10].

Early reports isoniazid and rifampicin mono-resistance have been presumed to have minimal clinical impact, causing it to be a topic of debate [1, 2, 11, 12]. A meta-analysis demonstrated a strong association between initial isoniazid mono-resistance and treatment failure [9]. There have also been concerns about rifampicin mono-resistant patients acquiring multidrug resistance, specifically in populations where resistance is frequent [13]. Studies have noted previous TB treatment as isoniazid mono-resistant risk factors [14, 15]. Due to few rifampicin mono-resistant patients, risk factors have not been well characterized; but prior studies have identified prior TB treatment and HIV co-infection [1, 16, 17].

Treatment outcomes of isoniazid mono-resistant TB have been well defined in countries with high HIV prevalence, but there is limited data from low HIV prevalent regions [2, 5, 8]. Minimal studies have also been focused on rifampicin mono-resistance in both high and low HIV prevalent countries. Our study addressed the prevalence, risk factors, and treatment outcomes associated with isoniazid and rifampicin mono-resistance among persons diagnosed with their first episode of culture-confirmed pulmonary TB in a high TB incidence and low HIV prevalence district in Lima, Peru.

## Methods

### Study Setting

The study was conducted in San Juan de Lurigancho (SJL), a densely-populated district of Lima, Peru (population: 1,069,566 persons) representing 3.5% of the Peruvian population [18]. It is one of the poorest districts in Peru, and contains one of the largest prisons in the country. The SJL district has 33 health care centers and one referral hospital managed by the Ministry of Health, with each site having a TB unit to provide TB treatment under directly observed therapy short-course (DOTS) following National TB Program (NTP) guidelines; the study was conducted throughout the 34 facilities within the district of SJL. In 2007, SJL reported 7.0% (2,004/29,393) of all TB cases notified to the National TB Program (NTP) and 14.2% (116/818) of the MDR-TB cases [19].

### Study Design

We performed a prospective observational cohort study among adults with a smear-positive pulmonary TB diagnosed between March 2010 and December 2011 with no history of previous TB treatment or diagnoses. Patients initiated anti-TB treatment at the NTP site and were followed through the end of their treatment regimen. Treatment outcomes were prospectively obtained from the NTP registers (see outcome definitions below). TB registers were monitored monthly up to two years after the end of treatment of the last enrolled case for TB recurrence. If a recurrent episode was found among an enrolled and cured TB patient, a sputum sample was gathered if available.

We ascertained demographic, epidemiological, and clinical characteristics of all study participants through interviews using a structured questionnaire. Participants were asked about their education, use and type of general public transportation, employment, prison exposure, illicit drug use, MDR-TB contacts, and comorbidities. The CAGE alcoholism-screening test was used to determine if a patient is at low or high risk of alcohol abuse [20]. It is a standardized four-question test that has been extensively validated as a screening technique. Socioeconomic status (SES) was determined by a scale validated by the Peruvian Ministry of Finance to determine poverty, extreme poverty, and non-poverty in urban and rural households (SISFOH)[21]. All patients were offered HIV screening as part of their routine NTP care. We registered the results of those tested.

### TB diagnostic laboratory tests

A single sputum specimen for each patient was collected at diagnosis. Sputa were cultured on Löwenstein-Jensen (LJ) media at the Tuberculosis Laboratory of the Instituto de Medicina Tropical Alexander von Humboldt (Universidad Peruana Cayetano Heredia) in Lima, Peru. Drug susceptibility testing (DST) was performed using the 7H10 agar method with the following concentrations: Isoniazid low level (0.2 μg/ml), high level (1 μg/ml), and rifampicin (1.0 μg/ml). Patients were also recorded if they had a recurrent TB episode.

### Definitions

Isoniazid mono-resistance was defined as isoniazid resistant and rifampicin susceptible, while rifampicin mono-resistance included patients that were rifampicin resistant and isoniazid susceptible [4]. Ethambutol and Streptomycin resistance were also tested, but not utilized within this study. Isoniazid resistance was classified as low-level or high-level based on presence of growth of *M*. *tuberculosis* at 0.2 μg/ml or 1 μg/ml of isoniazid, respectively. Simultaneous resistance to isoniazid and rifampicin is defined as multidrug-resistant TB (MDR-TB) case. For the documented treatment regimens, the time frame in the table in S1 Table represents treatment start and end dates. Adherence was not measured. Treatment outcomes were based on World Health Organization classifications (cured + treatment completion, failure, lost to follow up, death, and transfer). The latter four were considered poor treatment outcomes. An additional category of “treatment change” was created because participants who were continuously smear-positive during their first four months of their regimen or did not improve clinically post treatment initiation, may have had a change in regimen. The patients physician and a decentralized expert committee from the NTP who review all drug resistant cases would have indicated this change based on clinical presentation or DST. With regards to the treatment regimens, standard regimen I was defined as initiating treatment with two months of isoniazid, rifampicin, ethambutol, and pyrazinamide, followed by four months of isoniazid and rifampicin. The other treatment regimens included medications from each of the five groups of second line drugs following national and international guidelines. Treatment changes were recorded for each patient. Recurrent TB episodes were assessed two years after completion of the treatment among cured patients.

### Data Management and Analysis

The data was entered in Microsoft Access (Microsoft Corporation, Redmond, WA, US) and statistical analyses were performed in STATA 13.1 for Mac (Stata Corporation, College Station, Texas). Bivariate analyses were conducted using the chi-squared and the Mann-Whitney rank-sum test for dichotomous and continuous variables, respectively. If the variables had a p-value of <0.2 in the univariate logistic regression, or were considered to be clinically important, they were included in a backward elimination method of logistic regression to generate isoniazid and rifampicin mono-resistance prediction models. Unadjusted and adjusted odds ratios (ORs) with 95% confidence intervals (CI) were also recorded. In addition, to determine the association between isoniazid and rifampicin mono-resistance and treatment outcomes a forward logistic regression analysis was performed, accounting for potential confounders.

### Ethical considerations

The cohort study was approved by the Institutional Review Boards at Universidad Peruana Cayetano Heredia, University of Antwerp and at the District Health Direction for East Lima. This subanalysis was also approved by the Vanderbilt University School of Medicine. All participants signed informed consent and were issued a copy of the signed consent form. DST results were given to each patient’s physician in each health facility, for patient management.

## Results

### Study Population

There were 1,292 persons enrolled in the cohort, of who 1039 (80%) had culture-confirmed TB. Among the 253 that were not confirmed: 115 (45.5%) were culture negative, 49 (19.4%) had a contaminated culture, 31 (12.3%) had an insufficient sample to allow growth, 11 (4.3%) were not processed, and 47 (18.6%) did not submit a sample. The enrolled patients who had culture-confirmed TB were similar to those that were culture negative except that the culture-confirmed TB patients were more likely to report current or former tobacco use and were less likely to have had an undergraduate or post-graduate education level.

### Drug Resistant Tuberculosis

There were 85/1039 (8.2%, 95%CI: 6.57–10.07) cases of isoniazid mono-resistant TB. Of these, 45 (53%) had high-level isoniazid resistance and 40 (47%) had low-level isoniazid resistance. Rifampicin mono-resistant TB was found in 24/1039 (2.3%, 95%CI 1.51–3.39) and MDR-TB in 69/1039, (6.6%, 95%CI 5.24–8.28). Table 1 demonstrates the characteristics of isoniazid mono-resistant, rifampicin mono-resistant, MDR-TB, and isoniazid and rifampicin susceptible cases. The MDR-TB group had higher proportion of males and reported a higher proportion of prior rehabilitation center placement.

**Table 1:** Demographic and Clinical Characteristics of Isoniazid and Rifampicin Susceptible, Isoniazid Mono-resistant, Rifampicin Mono-resistant, and Multidrug-Resistant Pulmonary Tuberculosis Patients.

	Isoniazid &		Rifampicin		Isoniazid			
	Rifampicin Susceptible		Mono-resistance		Mono-resistance		Multi-Drug Resistance	
	n = 861		n = 24		n = 85		n = 69	
	n	%	n	%	n	%	n	%
**Age**								
<40	674	78.3	19	79.2	70	82.4	56	81.2
>40	187	21.7	5	20.8	15	17.5	13	18.8
**Sex**								
Male	528	61.3	16	66.7	49	57.6	53	76.8
Female	333	38.7	8	33.3	36	42.4	16	23.2
**Use of public transportation**								
Small/large bus	629	73.1	17	70.8	61	71.8	47	68.1
Taxi/Shared taxi	189	22.0	7	29.2	20	23.5	17	24.6
Does not use	43	5.0	0	0	4	4.7	5	7.2
**Tobacco use**								
Never	506	58.8	12	50	48	56.5	36	52.2
Former/current	353	41.0	12	50	37	43.5	33	47.8
**Alcoholism**								
0–1 (low suspicion)	645	74.9	17	70.8	71	83.5	48	69.6
2–4 (high suspicion)	184	21.4	7	29.2	12	14.1	19	27.5
**Illicit drug use**								
No	723	84.0	20	83.3	67	78.8	51	73.9
Yes	138	16.0	4	16.7	18	21.2	18	26.1
**Past rehabilitation center admission**								
No	800	92.9	23	95.8	78	91.8	61	88.4
Yes	60	7	0	0	7	8.2	8	11.6
**MDR contact**								
No/unknown	804	93.4	24	100	78	91.8	58	
Yes	57	6.6	0	0	7	8.2	11	15.9
**HIV status**								
Negative	623	72.4	13	54.2	58	68.2	56	81.2
Sero-positive	11	1.3	2	8.3	3	3.5	4	5.8
Unknown	227	26.4	9	37.5	24	28.2	9	13
**Prior use of isoniazid prophylaxis**								
No	842	97.8	23	95.8	83	97.6	66	95.7
Yes	18	2.1	0	0	2	2.4	2	2.9
**Socioeconomic status**								
No poverty	595	69.1	18	75	51	60	43	62.3
Poverty	210	24.4	5	20.8	28	32.9	21	30.4
**Prior Imprisonment**								
No	820	95.2	21	87.5	83	97.6	65	94.2
Yes	40	4.6	3	12.5	2	2.4	4	5.8

### Characteristics Associated to Isoniazid and Rifampicin Mono-resistance

The bivariate and multivariate analyses for characteristics associated with isoniazid and rifampicin mono-resistance are shown in Tables 2 and 3, respectively. In the multivariable analysis of isoniazid mono-resistant individuals, the best prediction model consisted of HIV status, illicit drug use, prior imprisonment, alcoholism, socioeconomic status, and type of transportation. The report of illicit drug use was the only factor significantly associated (aOR 2.06, 95% CI 1.1–4.1) with isoniazid mono-resistance. In the multivariate analysis of rifampin mono-resistance, the HIV positive status was the only factor found to be weakly associated with mono-resistance (aOR 9.43, 95% CI 1.9–47.8), however, this was based on only two patients.

**Table 2:** Multivariate Analysis of Characteristics Associated with Isoniazid Mono-resistance.

Variables	Isoniazid Mono-resistance		Isoniazid and Rifampicin Susceptible		Unadjusted Odds Ratio [95%CI]	Adjusted Odds Ratio [95%CI]
	n	%	n	%		
**Age**						
<40	70	82.3	681	79.1		
>40	15	17.7	180	20.9	0.71 [0.4–1.3]	-
**Sex**						
Male	49	57.6	528	61.3	0.91 [0.6–1.4]	-
Female	36	42.4	333	38.7		
**Diabetes**						
No	82	96.5	825	95.8		
Yes	3	3.5	36	4.1	0.83 [0.3–2.8]	-
**Use of public transportation**						
Small/large bus	61	71.8	629	73.1	3.91 [0.5–29.0]	4.06 [0.5–30.3]
Taxi/shared taxi	20	23.5	189	22.0	4.40 [0.6–34.0]	4.49 [0.6–34.8]
Does not use	4	4.7	43	5.0		
**Tobacco use**						
Never	48	56.5	506	58.8		
Former/current	37	43.5	353	41.0	1.17 [0.7–1.9]	-
**Alcoholism**						
0–1 (low suspicion)	71	83.5	645	74.9		
2–4 (high suspicion)	12	14.1	184	21.4	0.60 [0.3–1.2]	0.52 [0.3–1.1]
**Illicit drug use**						
No	67	78.8	723	84.0		
Yes	18	21.2	138	16.0	1.48 [0.8–2.7]	2.10 [1.1–4.1]
**Past rehabilitation center admission**						
No	78	91.8	800	92.9		
Yes	7	8.2	60	7.0	1.37 [0.6–3.3]	-
**MDR contact**						
No/unknown	78	91.8	804	93.4		
Yes	7	8.2	57	6.6	1.38 [0.6–3.2]	-
**HIV status**						
Negative	58	68.2	623	72.4		
Sero-positive	3	3.5	11	1.3	3.20 [0.9–12.0]	2.75 [0.7–10.8]
Unknown	24	28.2	227	26.4	1.23 [0.7–2.0]	1.17 [0.7–2.0]
**Prior use of isoniazid prophylaxis**						
No	83	97.6	843	97.9		
Yes	2	2.4	18	2.1	1.09 [0.2–4.8]	-
**Socioeconomic status**						
No poverty	51	60	595	69.1		
Poverty	28	32.9	210	24.4	1.53 [0.9–2.5]	1.53 [0.9–2.5]
**Prior imprisonment**						
No	83	97.6	821	95.4		
Yes	2	2.4	40	4.6	0.29 [0.04–2.1]	0.24 [0.03–1.9]

**Table 3:** Multivariate Analysis of Characteristics Associated with Rifampicin Mono-resistance.

Variables	Rifampicin Mono-resistance		Isoniazid and Rifampicin Susceptible		Unadjusted Odds Ratio [95%CI]	Adjusted Odds Ratio [95%CI]
	n	%	n	%		
**Age**						
<40	19	82.6	681	79.1		
>40	4	17.4	180	20.9	0.75 [0.3–2.2]	-
**Sex**						
Male	16	66.7	528	61.3	1.28 [0.5–3.1]	
Female	8	33.3	333	38.7		
**Use of public transportation**						
Small/large bus	17	70.8	629	73.1	0.65 [0.3–1.6]	-
Taxi/shared taxi	7	29.2	189	22.0		
Does not use	0	0.0	43	5.0		
**Tobacco**						
Never	12	50.0	506	58.8		
Former/Current	12	50.0	353	41.0	1.42 [0.6–3.3]	-
**Alcoholism**						
0–1 (low suspicion)	17	70.8	645	74.9		
2–4 (high suspicion)	7	29.2	184	21.4	1.31 [0.5–3.4]	-
**Illicit drug use**						
No	20	83.3	723	84.0		
Yes	4	16.7	138	16.0	1.33 [0.4–4.0]	-
**HIV status**						
Negative	13	54.2	623	72.4		
Sero-positive	2	8.3	11	1.3	9.43 [1.9–47.8]	9.43 [1.9–47.8]
Unknown	9	37.5	227	26.4	2.13 [0.9–5.1]	2.13 [0.9–5.1]
**Socioeconomic status**						
No poverty	18	75.0	595	69.1		
Poverty	5	20.8	210	24.4	0.78 [0.3–2.1]	-
**Prior imprisonment**						
No	21	87.5	821	95.4		
Yes	3	12.5	40	4.6	2.14 [0.5–9.5]	-

### Treatment Regimens and Outcomes

The primary endpoint was the outcome at the end of treatment. This analysis demonstrated that isoniazid-mono-resistant patients were more likely to die (4/85, 5%, p = 0.014) and to fail treatment (2/85, 2%, p<0.01) compared to persons with isoniazid-susceptible TB (Table 4). Rifampicin mono-resistant patients also had an increased risk of death (2/24, 8%, p<0.01), as shown in Table 4, when compared to rifampicin-susceptible TB cases. The proportion of high level and low level isoniazid mono-resistant patients who were cured are 76% and 73%, respectively, with minor differences in the poor outcome categories.

**Table 4:** End of Treatment Outcomes for Isoniazid and Rifampicin Mono-resistant TB Cases.

	Isoniazid Mono-resistance			Rifampicin Mono-resistance			Isoniazid & Rifampicin Susceptible	
	n	%	p- value	n	%	p-value	n	%
**Good outcome**								
Cured	63	74.1	0.006	17	70.8	0.049	735	85.4
**Poor outcome**								
Death	4	4.7	0.014	2	8.3	0.004	12	1.4
Failure	2	2.4	0.009	-	-	-	3	0.3
Loss to follow up	13	15.3	0.084	4	16.7	0.217	87	10.1
Transfer	3	3.5	0.545	1	4.2	0.570	24	2.8

Additionally, poor treatment outcomes were more frequent among the isoniazid mono-resistant population than in the isoniazid and rifampicin sensitive group (22/85, 26% vs. 126/861, 15%, p<0.01) when compared to the successfully treated patients. The rifampicin mono-resistant subgroup had a higher risk of poor treatment outcomes than the isoniazid and rifampicin sensitive group (7/24, 29% vs. 126/861, 15%, p<0.05).

Standard regimen I was started in 78 isoniazid-mono-resistant patients, of which 36 patients completed the course of six months and 33 patients were switched to a drug resistant regimen detailed in the table in S1 Table. Standard regimen I was initiated in all the rifampicin mono-resistant patients, but three cases were subsequently changed to a drug resistant treatment noted in the table in S2 Table. Sixteen out of the 17 total cured rifampicin mono-resistance patients were cured with standard regimen I. A review of those cases, found that two of them were found to be rifampicin susceptible in a different DST from a routine sputum sample submitted within two days of the study sample. Of the 17 cured rifampicin mono-resistant patients, six were followed up within two years after cure, and seven were followed up to one year after cure. In all cases patients reported to be asymptomatic during that period.

None of the isoniazid mono-resistant participants that were cured (63/85, 74%) had a documented recurrent episode of TB. Among the rifampicin mono-resistant participants who were cured (17/24, 71%), one had a documented reinfection confirmed with spoligotyping and MIRU-VNTR analysis (1/17, 6%), one had a recurrent episode that was not further classified (1/17, 6%), and fifteen had no documented recurrent episodes (15/24, 88%) during the two year follow-up.

## Discussion

This study, the largest prospective cohort on isoniazid and rifampin mono-resistant TB to date, found high proportions of isoniazid and rifampicin mono-resistance in a setting of low HIV prevalence. Twenty-six percent of the patients with rifampicin resistance (24/93) were found to not be MDR-TB. Illicit drug use was a risk factor for isoniazid mono-resistance, while HIV infection was associated with rifampicin mono-resistance. Deaths and treatment failures were more frequent among patients with isoniazid mono-resistance and deaths were more frequent among rifampicin mono-resistant patients. Meanwhile, high-level and low-level of isoniazid mono-resistance had similar treatment outcomes. The patients within our study were treated with diverse regimens; however, high cure rates were encountered with rifampicin, ethambutol, pyrazinamide, and the addition of levofloxacin.

Primary drug resistance has been increasing with isoniazid mono-resistance being the largest population particularly in the high burden regions [22]. The global prevalence of isoniazid monoresistance has been estimated at 8.1% for new TB cases with a higher percentage in the coinfected-HIV population [4, 23]. We found a similar prevalence in a low HIV burden setting. Findings from the same study district in Lima suggest ongoing transmission of drug resistant strains [24, 25]. There are limited studies on rifampicin mono-resistance; however, Western Europe reported <0.3%, while proportions as high as 1.3% have been reported in Mexico and Zambia [6, 22]. Our study found a relatively high prevalence: 2.3%, however our definition was less stringent than other studies, because we defined mono-resistance solely based on isoniazid and rifampicin without including the other first line drugs. Rifampicin resistance has frequently been considered a proxy for MDR. Our results call for careful evaluation of implementation of DSTs that only test for rifampicin resistance which may result in giving MDR treatment to isoniazid susceptible patients.

Past studies have demonstrated a strong correlation between history of TB treatment with isoniazid mono-resistance [2, 15, 16, 26]. However, none of our patients received prior TB treatment since we were specifically evaluating primary resistance. Our study contained two patients with isoniazid monoresistance that reported prior isoniazid prophylaxis, but illicit drug use was the only significant factor within the prediction model. Previous studies have shown rifampicin mono-resistance associated with history of TB, prior imprisonment, and alcohol abuse [12, 17]. Our study confirmed a weak association between HIV co-infection and rifampicin mono-resistance [1, 3]. We hypothesize that since Peru has a low burden of HIV, HIV care is centralized, this population may be at higher risk of exposure to drug resistant TB due to their frequent attendance at specialized medical facilities.

Isoniazid mono-resistance treatment outcomes have been a topic of debate due to conflicting studies. Our results were compatible with prior reports demonstrating that isoniazid mono-resistant cases are at a higher risk for poor outcomes, specifically failures and deaths. We found no differences in treatment outcomes between the lower and higher concentrations of isoniazid resistance similar to the findings of Espinal et al and Chien et al [8, 27]. A significant association suggested that the rifampicin mono-resistant population was at higher risk of death, however this was only based on two patients and should be evaluated in a larger sample. One study in France that included 39 cases reported that only 67% were cured. In regards to long-term outcomes, our study found that none of the isoniazid mono-resistant and one of the rifampicin mono-resistant cases had a recurrence, which could not be tested to determine if it was a relapse or a reinfection; a larger population would be more informative to determine the impact of mono-resistance on TB relapse. There are standardized regimens for isoniazid mono-resistant patients and recommendations for the rifampicin mono-resistant population; however, there is limited evidence supporting their benefit. Our study highlighted how poor outcomes may be more frequent with mono-resistant TB emphasizing the need for appropriate treatment regimens and outreach required to apply them into communities.

As this was an observational study, there were limitations inherent to the study design. First, we had missing information for several of our cases, specifically alcoholism and socioeconomic status, causing our sample size to be decreased. Second, the rifampicin mono-resistant sample size was a small subgroup, generating insufficient statistical power to be able to fully address association with risk factors and treatment outcomes. Third, our long-term outcomes only provided information for cured patients, and in many cases it was only a passive follow up. This may have underestimated relapses, which would over estimate long-term success. The study also used mono-resistance based on isoniazid and rifampicin susceptibilities.

In conclusion, isoniazid and rifampicin mono-resistance were frequent in our setting and were associated with an increased risk of death. The knowledge gaps that still need to be addressed includes proper treatment regimens and improvement of accessibility for the patients to provide better outcomes [22]. The importance of facilitating second-line regimens in a DOTS program when drug resistant TB strains are detected is crucial based on the high risk of developing MDR-TB with standard short-course chemotherapy and propagating drug-resistant TB [28].

## Supporting Information

S1 Table Treatment Regimens and Outcomes in Isoniazid Mono-resistant Cases (N = 85). (DOCX)

S2 Table Treatment Regimens and Outcomes in Rifampicin Mono-resistant Cases (N = 24). (DOCX)
