# Supplementary material for: Prevalence, Risk Factors, and Treatment Outcomes of Isoniazid- and Rifampicin- Mono-Resistant Pulmonary Tuberculosis in Lima, Peru
Source: PLoS One. 2016 Apr 5;11(4):e0152933. doi: 10.1371/journal.pone.0152933 (PMC4821555; doi:10.1371/journal.pone.0152933)
Supplement: S1 Table — (DOCX) [file pone.0152933.s001.docx]

| Treatment Regimen | | | N (%) |
| --- | --- | --- | --- |
|  |  |  |  |
| 6 months (N=52) | |  |  |
| 2HREZ/4HR |  |  | 52 (100%) |
| Did not complete | |  |  |
| Death |  |  | 4 (8%) |
| Default | |  | 9 (17%) |
| Transfer | |  | 2 (4%) |
| Failure | |  | 1 (2%) |
| Completed | |  |  |
| Cured |  |  | 36 (69%) |
|  |  |  |  |
| 7-12 months (N=21) | |  |  |
| REZ |  |  |  |
| Cured |  |  | 2 (10%) |
| REZLx |  |  |  |
| Unknown | |  | 1 (5%) |
| Cured |  |  | 2 (10%) |
| Default | |  | 1 (5%) |
| HREZ/DrugRes | |  |  |
| Cured |  |  | 1 (5%) |
| 2HREZ/REZ |  |  |  |
| Cured |  |  | 1 (5%) |
| 2HREZ/RE |  |  |  |
| Cured |  |  | 1 (5%) |
| 2HREZ/4HR/extHR | |  |  |
| Cured |  |  | 1 (5%) |
| 2HREZ/HR/REZLx | |  |  |
| Cured |  |  | 10 (48%) |
| 2HREZ/HR/RELxK | |  |  |
| Default | |  | 1 (5%) |
|  |  |  |  |
| >1 year (N=9) | |  |  |
| DrugRes |  |  |  |
| Cured |  |  | 1 (11%) |
| HREZ/DrugRes | |  |  |
| Cured |  |  | 1 (11%) |
| 2HREZ/HR/REZ | |  |  |
| Cured |  |  | 1 (11%) |
| 2HREZ/HR/REZLx | |  |  |
| Cured |  |  | 4 (44%) |
| Transfer | |  | 1 (11%) |
| 2HREZ/HR/REZCx | |  |  |
| Cured |  |  | 1 (11%) |
|  |  |  |  |
| Unknown months (N=3) | |  |  |
| 2HREZ/HR/DrugRes | |  |  |
| Default | |  | 1 (33%) |
| Cured |  |  | 1 (33%) |
| Failure | |  | 1 (33%) |

H, isoniazid; R, rifampicin; E, ethambutol; Z, pyrazinamide; Lx, levofloxacin; Cx, ciprofloxacin; ExtHR, extended isoniazid & rifampicin; DrugRes, drug resistant.

**S1 Table. Treatment Regimens and Outcomes in Isoniazid Mono-resistant Cases (N = 85)**
