# Supplementary material for: Prevalence, Risk Factors, and Treatment Outcomes of Isoniazid- and Rifampicin- Mono-Resistant Pulmonary Tuberculosis in Lima, Peru
Source: PLoS One. 2016 Apr 5;11(4):e0152933. doi: 10.1371/journal.pone.0152933 (PMC4821555; doi:10.1371/journal.pone.0152933)
Supplement: S2 Table — (DOCX) [file pone.0152933.s002.docx]

| Approximate Treatment Regimen | | | N (%) | |
| --- | --- | --- | --- | --- |
|  |  |  | |  |
| 6 months (N=23) |  |  | |  |
| 2HREZ/4HR |  |  | | 21 (88%) |
| Did not complete |  |  | |  |
| Death |  |  | | 1 (5%) |
| Default |  |  | | 3 (14%) |
| Transfer |  |  | | 1 (5%) |
| Completed |  |  | |  |
| Cured |  |  | | 16 (76%) |
|  |  |  | |  |
| 2HREZ/2Other |  |  | |  |
| Death |  |  | | 1 (4%) |
| 2HREZ/4Other |  |  | |  |
| Default |  |  | | 1 (4%) |
|  |  |  | |  |
| 7-12 months (N=1) |  |  | |  |
| 2HREZ/2HR/5DrugRes |  |  | |  |
| Cured |  |  | | 1 (4%) |

H, isoniazid; R, rifampicin; E, ethambutol; Z, pyrazinamide; Other, drug resistant regimen.

**S2 Table. Treatment Regimens and Outcomes in Rifampicin Mono-resistant Cases (N = 24)**
